# Destinations fostering older adults’ walking for transport: a cross-sectional study from Germany

**DOI:** 10.1186/s12877-022-02896-w

**Published:** 2022-03-17

**Authors:** Pia Hasselder, Tanja Brüchert, Sabine Baumgart, Gabriele Bolte

**Affiliations:** 1 University of Bremen, Institute of Public Health and Nursing Research, Department of Social Epidemiology, Grazer Str. 4, 28359 Bremen, Germany; 2 Health Sciences Bremen, University of Bremen, 28359 Bremen, Germany; 3 ARL, Academy for Spatial Development in the Leibniz Association, Hannover, Germany

**Keywords:** Active mobility, Walking for transport, Built environment, Destinations, Neighborhood, Older adults, Active aging, Healthy aging, Age-friendly environment

## Abstract

**Background:**

Having destinations within walking distance can encourage older people to walk. Yet, not all destinations may be equally important. Little is known about the types of destinations fostering older adults’ walking for transport in small and medium-sized towns and rural communities. The aim of this study was to explore the associations between the availability of different destinations and walking for transport among older adults living in communities with less than 100,000 inhabitants.

**Methods:**

Between May and September 2019, self-reported data from 2242 older adults (≥ 65 years) living in the Metropolitan Region Northwest (Germany) were collected within the project *AFOOT – Securing urban mobility of an aging population*. Data from 2137 study participants were eligible for this analysis. Logistic regression models were used to investigate the relationship between the perceived destination availability of 19 different destinations within a 20-min or 10-min walk from home, respectively, and the engagement in walking for transport. Crude and adjusted models were run separately for each destination and distance category. Exploratory subgroup analyses examined the associations between the availability of destinations within a 20-min walk from home and walking for transport stratified by gender, use of a walking aid, and car availability.

**Results:**

The availability of each of the investigated destinations within a 20-min walk and of nearly all of these destinations within a 10-min walk from home was significantly positively associated with walking for transport in crude models. Most associations remained significant after adjustment for covariates. The strongest associations were found for the availability of small stores, pharmacy, and bakery. The availability of a bus stop showed the weakest associations and was not significantly associated with walking for transport after adjustment for covariates.

**Conclusions:**

The provision of local amenities within walking distance may be a promising approach to foster older adults’ walking for transport in smaller communities with less than 100,000 inhabitants and to enable active and healthy aging in place. Further quantitative and qualitative research is needed to validate these findings and to better understand older adults’ walking behavior.

**Supplementary Information:**

The online version contains supplementary material available at 10.1186/s12877-022-02896-w.

## Background

The health needs of a rising number and proportion of older adults poses a major public health challenge to many countries in the world [1]. This is especially true for Germany which is one of the countries with the highest share of older adults in the European Union [2]. In 2019, already 22% of the total population was aged 65 years and older [3].

In response to an aging population, the World Health Organization published a policy framework for active aging in 2002 [4] and a world report on aging and health in 2015 [5]. Based on this, the United Nations most recently declared 2021–2030 the Decade of Healthy Ageing to boost global action to promote active and healthy aging worldwide [6].

A key approach is the promotion of physical activity until old age [7, 8]. Engaging in regular physical activity has many health benefits for older adults including reduced risks of chronic diseases and all-cause mortality [9]. Furthermore, engagement in physical activity is important to maintain independence in pre-frail and frail older adults [10].

Despite the widely known health benefits, many older adults are physically inactive [11]. In Germany, only 38% of older adults currently meet the WHO recommendation of 150 min of moderate-to-vigorous physical activity per week [12]. However, the prevalence of walking for transport increases in older age groups and walking for transport seems to be the most accessible mode of transport for older adults until very old age [13]. Walking for transport is a promising opportunity to integrate regular moderate physical activity in daily routines and thereby to enhance physical activity levels in this age group.

Former research has shown that several attributes of the adjacent built environment are associated with the engagement in walking for transport among older adults [14]. The most consistent evidence has been found for the availability of destinations and services which has been identified as one of the most important attributes of the built environment for older adults’ walking for transport [14]. Providing nearby destinations may encourage older adults to walk to these destinations and thereby integrate regular physical activity in their day-to-day trips.

However, little is known about the specific types of destinations fostering older adults’ walking for transport [15, 16]. Most evidence exists for the positive associations of commercial destinations [17–19], public transport stops [20–23] and recreational destinations [20, 24–26] with total walking for transport. There are also a few studies showing a positive relationship between the availability of other types of destinations such as food outlets and walking for transport [14].

It should be noted that most of these former studies have been carried out in large cities and almost all studies focusing on specific types of destinations have been conducted outside Europe (e.g., Hong Kong, US, Canada) [14]. Due to different settlement patterns regarding density and land use mix the results of these former studies are not easily transferable to our study region. Consequently, there is a lack of studies investigating the association of specific types of destinations with walking for transport among older adults living in small and medium-sized towns and rural communities in the European context.

Additionally, most studies analyzed associations between destinations and walking for transport only in the overall population of older adults. To better reflect the great diversity and heterogeneity of older adults and their mobility behavior, it is important to examine these associations also in subgroups of older adults: There is some evidence that more women than men engage in walking for errands [27] and a Japanese study suggests that associations between the availability of specific destinations and walking for transport may differ by gender [19]. Examining gender-specific associations appears also highly relevant in the context of gender planning. In spatial planning, gender aspects in terms of different mobility patterns, requirements for the design and utility value of public spaces, and different patterns of spatial appropriation are increasingly taken into consideration. Additionally, a short distance to destinations may be more relevant for people using a walking aid. Furthermore, people who have always a car available have different mobility options than people who have limited or no access to a car. Thus, the associations between the availability of destinations and walking for transport may also differ by car availability.

The aim of this study is to explore the relationship between the availability of different destinations within walking distance and older adults’ walking for transport in communities with less than 100,000 inhabitants in Northern Germany. Additionally, this study examines these associations in subgroups of gender, use of a walking aid, and car availability. The findings may provide further information on how to develop activity-friendly environments for an aging population.

## Methods

### Study design

A cross-sectional study was carried out from May to September 2019 among older adults (≥ 65 years) in the Metropolitan Region Northwest, Germany. This study was organized within the project *AFOOT – Securing urban mobility of an aging population* [28] and conducted in the form of a postal survey. The overall goal of the survey was to learn more about the mobility behavior of older adults living in small and medium-sized towns and rural communities. Ethical approval was provided by the University of Bremen ethical committee (ethics vote 20181205).

### Study population and setting

The study population was composed of older adults aged 65 and over living in rural communities or small and medium-sized towns with less than 100,000 inhabitants in the Metropolitan Region Northwest. The study region included 11 rural districts and two urban municipalities.

### Data collection

A sample of 11,000 older adults aged 65 years and older was randomly selected among the respective age group by the 117 residents’ registration offices of the study region. The number of adults drawn per registration office was based on the demographic structure of the corresponding spatial units, i.e., a higher proportion of older adults in a specific district or municipality led to a higher number of people drawn for this spatial unit.

The AFOOT project team contacted the study population by sending a set of documents (cover letter, self-administered questionnaire, document for the declaration of consent and a prepaid envelope) to each person of the sample. The cover letter summarized the study aims and explained the data processing. The questionnaire and the consent declaration were provided with the request to fill them out and send them back to the University of Bremen. There was no reminder and participants did not receive any incentive. The total response rate was 20.6%.

Data from 2137 participants were eligible for this analysis. Participants were excluded from this analysis if they lived in an institution (*n* = 49) or had missing data on all exposure (*n* = 24) or outcome measures (*n* = 32).

### Measurement instrument

The AFOOT project team created a questionnaire with 49 questions, including aspects related to the neighborhood environment and mobility as well as health and sociodemographic characteristics of the participants. Most of the questions were worded according to validated instruments from national or international research (see references below). Focus group discussions with older adults (≥ 65 years) were used to pretest the questionnaire.

### Outcome

*Walking for transport* was defined as walking to get to and from destinations, e.g., to run errands, to commute to work or visit friends or family. Participants were asked “Do you walk for transport with a duration of at least 5 minutes?” According to their answer, participants were either classified into the category ‘Yes’ or ‘No’.

### Exposure

Based on the widely used Neighborhood Environment Walkability Scale (NEWS) [29, 30], the *availability of destinations* was assessed by asking participants to estimate the walking time from home to different types of destinations. A total of 19 different destinations which are common in Germany were listed. These included commercial destinations of daily needs (bakery, small grocery store, supermarket, drugstore, small stores), service providers (post office, bank/credit union, salon/barber shop, laundry/cleaner’s), eating places (café, restaurant), health services (physician, pharmacy), recreational destinations (recreation center, library, gym/fitness facility, park, cemetery), and public transport (bus stop). Participants could indicate the perceived distance on a 4-point scale (‘1–10 min’, ‘11–20 min’, ‘21–30 min’ and ‘31+ minutes’). The original responses on each item were categorized in two ways: Firstly, the responses were summarized as the availability within a 20-min walk from home (≤ 20 min vs. > 20 min), and secondly, as the availability within a 10-min walk from home (≤ 10 min vs. > 10 min).

### Covariates

#### Sociodemographic variables

*Gender* was assessed by asking participants to classify themselves as ‘Male’ or ‘Female’.

The *age* of the participants was derived from their reported year of birth and grouped into four categories according to quartiles (‘65–69 years’, ‘70–74 years’, ‘75–79 years’, ‘80 years and over’).

*Education* was described as a composite measure of school and professional education referring to the International Standard Classification of Education (ISCED) [31]. It was distinguished between three levels of education: ‘Low’ (ISCED 0–2), ‘Middle’ (ISCED 3–4), and ‘High’ (ISCED 5–6).

The monthly net income [32] and the number and age of household members were recorded to calculate the equivalized disposable *income* [33]. Based on the poverty line (60% of median income) of Lower Saxony [34], participants were grouped into three income categories: ‘Low’ (< 60% of median income), ‘Middle’ (≥ 60% of median income and ≤ median income), and ‘High’ (> median income).

Furthermore, participants were asked to report their *country of birth* [32]. Responses were divided into the categories ‘Germany’ and ‘Other country’.

Information on the relationship status [32] were recoded into the variable *partner status* denoting whether the participant had a partner (‘Partner’) or not (‘No partner’).

The *living situation* was derived from the reported number of people living together as an economic unit in the household [32]. Participants who answered with ‘1 person, i.e., only you’ were classified into the category ‘Living alone’, whereas those who indicated that they live together with at least one other person were considered as ‘Not living alone’.

The reported name of the municipality was used to determine the *area of residence* according to the classification of the Federal Institute for Research on Building, Urban Affairs and Spatial Development [35]. The respective categories were: ‘Medium-sized town’ (20,000–99,999 inhabitants), ‘Larger small town’ (10,000–19,999 inhabitants), ‘Small town’ (5000–9999 inhabitants or at least basic central function), and ‘Rural community’ (< 5000 inhabitants).

#### Health

*Self-rated health* was assessed by asking participants to rate their health on a 5-point scale (‘Very good’, ‘Good’, ‘Moderate’, ‘Poor’, ‘Very poor’) [36]. All response options are presented in Table 1. For all further analyses, a dichotomous variable was used (‘Very good, good’ and ‘Moderate, poor, very poor’).

**Table 1:** Characteristics of the study population

	Total (*n* = 2137)		Male (*n* = 1086)		Female (*n* = 979)	
	n	%	n	%	n	%
**Sociodemographics**						
Age, years						
65–69	585	28.3	299	27.6	286	29.2
70–74	531	25.7	270	24.9	260	26.6
75–79	494	23.9	264	24.4	230	23.5
80+	454	22.0	251	23.2	203	20.7
Education, ISCED						
Low	162	7.7	33	3.1	125	13.0
Middle	1201	57.1	529	49.5	623	64.6
High	741	35.2	507	47.4	217	22.5
Income						
Low	278	13.5	138	13.1	134	14.2
Middle	682	33.0	333	31.5	323	34.3
High	1107	53.6	585	55.4	485	51.5
Country of birth						
Germany	1982	96.5	1048	97.0	934	96.0
Other country	73	3.6	33	3.1	39	4.0
Partner status						
Partner	1607	78.0	930	85.8	676	69.3
No partner	454	22.0	154	14.2	300	30.7
Living situation						
Living alone	445	20.9	162	14.9	266	27.2
Not living alone	1686	79.1	922	85.1	711	72.8
Area of residence						
Medium sized town	641	30.3	310	28.9	315	32.5
Larger small town	776	36.7	385	35.9	360	37.2
Small town	533	25.2	291	27.1	223	23.0
Rural community	163	7.7	87	8.1	71	7.3
**Health**						
Self-rated health						
Very good	149	7.1	76	7.1	65	6.7
Good	1097	51.9	552	51.4	503	51.9
Moderate	716	33.9	357	33.2	340	35.1
Poor	143	6.8	86	8.0	55	5.7
Very poor	9	0.4	3	0.3	6	0.6
Mobility impairments						
At least one	800	38.0	404	37.5	381	39.7
None	1307	62.0	673	62.5	579	60.3
Use of a walking aid						
Yes	300	14.1	139	12.9	155	16.0
Walking stick/cane^a^	151	–	78	–	71	–
Crutches^a^	39	–	23	–	16	–
Walker^a^	170	–	65	–	102	–
Walking frame^a^	1	–	1	–	0	–
Quad walker^a^	0	–	0	–	0	–
Wheelchair^a^	37	–	15	–	21	–
No	1822	85.9	943	87.2	813	84.0
**Transport**						
Driving license						
Yes	1949	93.3	1037	97.2	847	88.7
No	141	6.8	30	2.8	108	11.3
Car availability						
Always	1869	89.4	1005	94.2	802	84.0
Sometimes	157	7.5	41	3.8	113	11.8
Never	65	3.1	21	2.0	40	4.2
Bicycle availability						
Yes	1804	84.9	957	88.5	778	80.0
No	321	15.1	124	11.5	194	20.0

^a^Multiple responses were possible, thus no relative frequencies are reported

Information about *mobility impairments* was gathered by asking participants whether they had a walking disability, a visual impairment or other mobility impairment(s). Multiple responses were possible. For this analysis, the original responses were collapsed into two categories: ‘At least one’ and ‘None’.

The *use of a walking aid* was also assessed by the questionnaire. Participants could answer with either ‘Yes’ or ‘No’. A ‘Yes’ could be completed with one or more of the following response options: ‘Walking stick/cane’, ‘Crutches’, ‘Walker’, Walking frame’, ‘Quad walker’, ‘Wheelchair’. The full range of listed walking aids is described in Table 1. For all further analyses, a dichotomous variable was used (‘Yes’/‘No’).

#### Transport

The ownership of a *driving license* was classified into the categories ‘Yes’ and ‘No’.

*Car availability* was explored by asking participants to indicate how often they can use a car as a driver or passenger. Three different response options were provided (‘Always’, ‘Sometimes’, and ‘Never’). All response options are reported in Table 1. For all further analyses, the original responses were recoded into two categories (‘Always’ and ‘Sometimes, never’).

*Bicycle availability* (‘Yes’/‘No’) was defined as the ownership of any type of bicycle (regular bicycle, electric bicycle/pedelec or tricycle).

#### Perceived neighborhood environmental attributes

*Walking infrastructure* and *connectivity* were assessed according to the Neighborhood Environment Walkability Scale (NEWS) [29, 30]. A list of statements about the neighborhood environment was provided and participants were asked to indicate how strongly they agree or disagree with these statements. The response options ranged from strongly disagree (coded as 1) to strongly agree (coded as 4). Subscale scores were calculated as the means of all respective items. Higher scores on these subscales indicate better walking infrastructure or connectivity.

### Data analysis

Statistical analyses were performed with SAS statistical software version 9.4 (SAS Institute Inc., Cary, NC, USA). Descriptive statistics (absolute and relative frequencies) were calculated for all abovementioned variables.

Logistic regression analyses were carried out to investigate the associations between the availability of destinations within walking distance from home (≤ 20 min and ≤ 10 min, respectively) and the engagement in walking for transport. Crude and adjusted models were run separately for each destination and distance category (≤ 20 min and ≤ 10 min). Adjusted models included gender, age, education, income, living situation, area of residence, use of a walking aid, bicycle availability, walking infrastructure, and connectivity as covariates. These covariates were chosen according to previous literature [14, 37]. Results of the logistic regression models are shown as Odds Ratios (OR) with corresponding 95% confidence intervals (CI). Confidence intervals were adjusted using the Bonferroni method [38]. According to this method, the significance level alpha = 0.05 was divided by the number of tests conducted. In this study, the associations between the perceived destination availability of 19 different destinations within walking distance and the engagement in walking for transport were investigated with 19 separate models (number of tests = 19). Thus, a *p*-value below 0.0026 (0.05/19 = 0.0026) for the single test was interpreted as statistically significant.

In exploratory subgroup analyses the associations between the availability of destinations within a 20-min walk from home and the engagement in walking for transport stratified by gender, use of a walking aid, and car availability were examined. Crude and adjusted logistic regression models were run separately for each destination and stratum. Adjusted models were run with the same covariates as in the main analysis. Due to the small size of the groups, it was not possible to further examine the associations between the availability of destinations within a 10-min walk from home and the engagement in walking for transport in subgroups of gender, use of a walking aid, and car availability.

In a sensitivity analysis, the main logistic regression analyses and the exploratory subgroup analyses stratified by gender and walking aid use were also run with further adjustment for car availability.

As a further exploratory approach, decision tree analyses were conducted to identify subgroups with a particularly high or low prevalence of walking for transport. These decision tree analyses were carried out in the form of CART analyses with R statistical software version 3.6.1 using the rpart package [39, 40]. Separate analyses were conducted for both distance categories in the overall study population and in subgroups of men and women.

## Results

### Study population characteristics

Table 1 provides an overview of the characteristics of the study population.

Study participants were 65 to 99 years old. The median age was 73 years (data not shown). The proportion of males (53%) was slightly higher than that of females (47%).

Overall, more than 90% of the study population had at least a middle level of education according to the International Classification of Education (ISCED). Nevertheless, the prevalence of a low educational level was four times higher in women than in men (13% vs. 3%). Fourteen percent of the total study population reported a low income as specified by the poverty threshold of the respective region (Lower Saxony).

Almost all participants were born in Germany (97%). About one third of women had no partner (31%) and more than a quarter lived alone (27%). In contrast, this applied to only 14 and 15% of men, respectively. Most of the participants lived in small and medium-sized towns. Only 8% of the study population lived in a rural community.

The majority of the study participants self-rated their health as good or very good but nearly 40% reported at least one mobility impairment and 14% the use of a walking aid. The walker and the walking stick/cane were by far the most common used walking aids. Women tended to use a walker more often than men. Men, in turn, tended to use a walking stick/cane and crutches more often than women.

Concerning transport aspects, the study population is characterized by a very high proportion of people holding a driving license (93%) and a high level of car and bicycle availability (89 and 85%). Nevertheless, results showed differences between men and women, with higher proportions among men than women across all transport aspects.

### Availability of destinations within a 20-min and a 10-min walk from home

Table 2 shows the perceived destination availability of 19 different destinations.

**Table 2:** Perceived destination availability within a 20-min and 10-min walk from home

	≤ 20 min.		> 20 min.		≤ 10 min.		> 10 min.	
	n	%	n	%	n	%	n	%
Commercial destinations								
Bakery	1485	69.5	652	30.5	872	40.8	1265	59.2
Small grocery store	1185	55.5	952	44.6	596	27.9	1541	72.1
Supermarket	1187	55.6	950	44.5	563	26.4	1574	73.7
Drugstore	679	31.8	1458	68.2	238	11.1	1899	88.9
Small stores	935	43.8	1202	56.3	330	15.4	1807	84.6
Service providers								
Post office	1122	52.5	1015	47.5	444	20.8	1693	79.2
Bank/credit union	1139	53.3	998	46.7	493	23.1	1644	76.9
Salon/barber shop	1222	57.2	915	42.8	591	27.7	1546	72.3
Laundry/cleaner’s	703	32.9	1434	67.1	235	11.0	1902	89.0
Eating places								
Café	1208	56.5	929	43.5	635	29.7	1502	70.3
Restaurant	1194	55.9	943	44.1	573	26.8	1564	73.2
Health services								
Physician	1126	52.7	1011	47.3	522	24.4	1615	75.6
Pharmacy	1176	55.0	961	45.0	547	25.6	1590	74.4
Recreational destinations								
Recreation center	1045	48.9	1092	51.1	383	17.9	1754	82.1
Library	805	37.7	1332	62.3	296	13.9	1841	86.2
Gym/fitness facility	1067	49.9	1070	50.1	432	20.2	1705	79.8
Park	1276	59.7	861	40.3	727	34.0	1410	66.0
Cemetery	1051	49.2	1086	50.8	444	20.8	1693	79.2
Public transport								
Bus stop	1676	78.4	461	21.6	1062	49.7	1075	50.3

Almost 80% of the study participants had a bus stop within 20 min walking distance, half of the participants even within 10 min walking distance from home. Thus, a bus stop was the most commonly available destination in both distance categories. The second and third most commonly available destinations were bakery and park. Seventy percent of the study participants lived within a 20-min walk of a bakery and 60% lived within this range of a park. A 10-min walk would take 41% of the study participants to a bakery and 34% to a park.

The least commonly available destinations within walking distance were library, laundry/cleaner’s, and drugstore. About a third of the participants lived within a 20-min walk of these destinations, slightly more than 10% even within a 10-min walk.

### Prevalence of walking for transport

Table 3 describes the prevalence of walking for transport in the overall population and stratified by gender, use of a walking aid, and car availability.

**Table 3:** Prevalence of walking for transport

	Any walking for transport				
	Yes		No		*p*-value^a^
	n	%	n	%	
Overall	1533	71.7	604	28.3	–
Stratified by gender					
Male	777	71.6	309	28.5	0.7414
Female	694	70.9	285	29.1	
Stratified by walking aid use					
Yes	164	54.7	136	45.3	<.0001
No	1358	74.5	464	25.5	
Stratified by car availability					
Always	1350	72.2	519	27.8	0.2387
Sometimes/never	152	68.5	70	31.5	

^a^*P*-value of chi-squared test

Overall, 72% of the study population indicated to engage in any walking for transport. The engagement in walking for transport did not differ by gender or car availability, but it differed between people using and not using a walking aid. Among people not using a walking aid, three out of four people reported to engage in any walking for transport. Among people using a walking aid, only half of the participants indicated to walk for transport.

### Associations between the availability of destinations and the engagement in walking for transport

Table 4 presents the results of the logistic regression analyses.

**Table 4:** Associations between the availability of destinations and walking for transport (*n* = 1746)

	Within a 20-min walk from home^a^				Within a 10-min walk from home^a^			
	Crude OR	95% CI^b^	Adj. OR^c^	95% CI^b^	Crude OR	95% CI^b^	Adj. OR^c^	95% CI^b^
Commercial destinations								
Bakery	2.62	1.86–3.69	1.95	1.30–2.91	2.44	1.72–3.47	1.87	1.28–2.74
Small grocery store	1.96	1.41–2.71	1.55	1.09–2.20	1.73	1.18–2.54	1.43	0.95–2.14
Supermarket	2.00	1.44–2.77	1.45	0.99–2.11	2.19	1.45–3.29	1.66	1.07–2.56
Drugstore	2.19	1.50–3.20	1.65	1.10–2.49	3.17	1.59–6.30	2.45	1.21–4.98
Small stores	2.68	1.89–3.79	2.21	1.51–3.22	2.89	1.65–5.05	2.25	1.26–4.01
Service providers								
Post office	2.29	1.64–3.18	1.82	1.27–2.62	2.30	1.46–3.64	1.82	1.13–2.93
Bank/credit union	2.21	1.59–3.06	1.73	1.21–2.49	2.37	1.53–3.68	1.90	1.20–3.02
Salon/barber shop	2.30	1.66–3.19	1.86	1.29–2.67	1.97	1.33–2.91	1.56	1.03–2.36
Laundry/cleaner’s	2.26	1.56–3.29	1.71	1.15–2.56	2.10	1.15–3.85	1.49	0.80–2.80
Eating Places								
Café	2.20	1.59–3.05	1.65	1.13–2.39	2.35	1.58–3.48	1.78	1.17–2.70
Restaurant	2.02	1.46–2.80	1.46	1.01–2.11	1.97	1.32–2.92	1.52	1.00–2.32*
Health services								
Physician	2.18	1.57–3.03	1.68	1.16–2.42	2.15	1.42–3.26	1.73	1.11–2.69
Pharmacy	2.46	1.77–3.43	1.97	1.36–2.85	2.62	1.71–4.01	2.09	1.34–3.28
Recreational destinations								
Recreation center	1.94	1.39–2.70	1.56	1.09–2.24	1.78	1.12–2.83	1.53	0.95–2.48
Library	2.28	1.59–3.26	1.90	1.29–2.80	2.30	1.31–4.04	1.86	1.03–3.36
Gym/fitness facility	1.90	1.37–2.64	1.49	1.04–2.13	1.71	1.11–2.63	1.34	0.85–2.11
Park	2.16	1.56–3.00	1.83	1.28–2.61	1.65	1.15–2.35	1.43	0.99–2.09
Cemetery	1.71	1.23–2.37	1.49	1.05–2.12	1.67	1.09–2.55	1.49	0.96–2.31
Public transport								
Bus stop	1.76	1.20–2.57	1.35	0.89–2.05	1.41	1.02–1.95	1.13	0.80–1.61

*OR* Odds Ratio, *CI* Confidence Interval, *Adj.* Adjusted

*After the Bonferroni adjustment the *p*-value was ≥0.0026 indicating a non-significant result

^a^Separate models were computed for all listed destinations

^b^Confidence intervals were adjusted using the Bonferroni method

^c^OR were adjusted for gender, age, education, income, living situation, area of residence, use of a walking aid, bicycle availability, walking infrastructure and connectivity

Overall, the availability of each of the listed destinations within a 20-min walking distance from the participants’ home was significantly positively associated with walking for transport in crude models and all but the supermarket and the bus stop also in adjusted models. The strongest associations with walking for transport were found for small stores, pharmacy, and bakery. After adjustment for covariates, the availability of each of these destinations within a 20-min walk from home approximately doubled the odds of engaging in walking for transport. The availability of a bus stop showed the weakest associations with walking for transport. It was the least strongly associated destination in crude models and did not remain significantly associated with walking for transport after adjustment for covariates.

Analyses examining associations between destinations within a 10-min walking distance from the participants’ home and walking for transport revealed quite similar results. Thus, all investigated destinations were significantly positively associated with walking for transport in crude models and most of the destinations even after adjustment for covariates. Small stores, pharmacy, and bakery were again among the destinations most strongly associated with walking for transport. Likewise, older adults were approximately two times more likely to engage in walking for transport when these destinations were present within a 10-min walk from home. However, the strongest association was found for the availability of a drugstore within a 10-min walk from home. The availability of a bus stop within a 10-min walking distance was not associated with walking for transport.

### Exploratory subgroup analyses

Table 5 reports the results of the gender-stratified logistic regression analyses.

**Table 5:** Associations between the availability of destinations and walking for transport, stratified by gender

	Men (*n* = 940)^a^				Women (*n* = 806)^a^			
	Crude OR	95% CI^b^	Adj. OR^c^	95% CI^b^	Crude OR	95% CI^b^	Adj. OR^c^	95% CI^b^
Commercial destinations								
Bakery	3.46	2.16–5.55	2.78	1.58–4.90	1.92	1.16–3.17	1.35	0.75–2.45
Small grocery store	2.20	1.40–3.44	1.74	1.06–2.83	1.71	1.06–2.76	1.35	0.81–2.25
Supermarket	2.37	1.51–3.71	1.74	1.03–2.94	1.65	1.03–2.66	1.19	0.69–2.05
Drugstore	2.25	1.34–3.75	1.64	0.94–2.87	2.12	1.21–3.72	1.64	0.89–3.00
Small stores	2.87	1.79–4.61	2.43	1.43–4.12	2.46	1.47–4.12	2.00	1.15–3.47
Service providers								
Post office	3.01	1.91–4.76	2.35	1.42–3.92	1.67	1.04–2.70	1.34	0.80–2.26
Bank/credit union	2.73	1.74–4.29	2.07	1.24–3.44	1.74	1.07–2.81	1.39	0.82–2.34
Salon/barber shop	2.79	1.77–4.39	2.36	1.40–3.99	1.85	1.15–2.99	1.48	0.87–2.49
Laundry/cleaner’s	2.75	1.62–4.68	2.16	1.22–3.82	1.83	1.07–3.12	1.37	0.77–2.43
Eating Places								
Café	2.74	1.74–4.31	2.24	1.33–3.76	1.72	1.07–2.77	1.24	0.71–2.15
Restaurant	2.66	1.69–4.17	2.00	1.20–3.36	1.48	0.92–2.39	1.05	0.61–1.80
Health services								
Physician	2.51	1.60–3.94	1.89	1.12–3.17	1.86	1.15–3.02	1.46	0.86–2.48
Pharmacy	2.71	1.73–4.27	2.16	1.29–3.62	2.21	1.36–3.57	1.75	1.03–3.00
Recreational destinations								
Recreation center	2.18	1.39–3.43	1.74	1.06–2.86	1.69	1.04–2.74	1.36	0.80–2.30
Library	2.51	1.54–4.08	2.10	1.22–3.60	2.02	1.19–3.45	1.67	0.94–2.97
Gym/fitness facility	2.40	1.53–3.78	1.80	1.09–2.98	1.46	0.90–2.35	1.20	0.72–2.01
Park	2.33	1.48–3.66	2.06	1.24–3.40	1.99	1.23–3.22	1.67	1.01–2.78
Cemetery	1.86	1.19–2.90	1.60	0.98–2.62	1.55	0.96–2.51	1.36	0.82–2.26
Public transport								
Bus stop	1.95	1.15–3.32	1.45	0.80–2.63	1.57	0.91–2.71	1.24	0.69–2.23

*OR* Odds Ratio, *CI* Confidence Interval, *Adj.* Adjusted

^a^Separate models were computed for all listed destinations

^b^Confidence intervals were adjusted using the Bonferroni method

^c^OR were adjusted for age, education, income, living situation, area of residence, use of a walking aid, bicycle availability, walking infrastructure and connectivity

Among men, the availability of each of the listed destinations within a 20-min walk was significantly positively associated with walking for transport in crude models and all but the drugstore, cemetery, and the bus stop also after adjustment for covariates. Among women, crude models also showed significant positive associations for nearly all listed destinations, but only small stores, pharmacy, and park did remain significantly positively associated with walking for transport after adjusting for covariates. However, it should be noted that all point estimates were in a positive direction.

Among men, it stands out that most destinations more than doubled the odds of engaging in walking for transport. The most strongly associated destinations were bakery, small stores, salon/barber shop, and post office. Among women, only the availability of small stores doubled the odds of engagement in walking for transport.

Results of the analyses restricted to people not using a walking aid showed that small stores, bakery, and pharmacy were the most strongly associated destinations within a 20-min walk from home. The weakest associations were found for the availability of a bus stop. Results of the analyses with people using a walking aid showed that none of the listed destinations were significantly associated with walking for transport after adjusting for covariates. However, results of this stratum must be treated with caution due to the small number of people using a walking aid and the high degree of uncertainty in these results (see Additional file 1: Table S1).

Among people having always access to a car, the availability of each of the listed destinations within a 20-min walk was significantly associated with higher odds of engagement in walking for transport in crude models and nearly all of these destinations did remain significantly associated with walking for transport after adjustment for covariates. The strongest associations were found for small stores and bakery. Analyses restricted to people with limited or no access to a car tended to result in considerably higher point estimates but also wider confidence intervals and more non-significant results. Again, these results must be treated with caution due to the small size of this group (see Additional file 1: Table S2).

In a sensitivity analysis, the results of the logistic regression analyses (see Table 4) and the exploratory subgroup analyses stratified by gender (see Table 5) and walking aid use (Table S1) remained stable after further adjustment for car availability.

### Decision tree analyses

The CART analyses did not reveal any subgroups with a particularly high or low prevalence of walking for transport that differ with respect to the availability of the destinations.

## Discussion

The aim of this study was to explore the relationship between the availability of different destinations within walking distance and older adults’ walking for transport in small and medium-sized towns and rural communities in Northern Germany and thereby identify destinations that are especially important for older adults’ walking for transport. Two different distance categories were used to define the availability of destinations to learn more about what is considered as a reachable walking distance for older adults.

Overall, this study showed that nearly all destinations were significantly positively associated with the engagement in walking for transport when they were reachable within a 20-min or a 10-min walk from home. In particular, the availability of almost each of the investigated commercial destinations, service providers, eating places, health services, and recreational destinations within a 20-min walk from home was significantly associated with higher odds of engaging in walking for transport. Most of the investigated commercial destinations, service providers, eating places and health services were also significantly associated with walking for transport when they were present within a 10-min walk from home. The availability of a bus stop within walking distance (≤ 20 min or ≤ 10 min) was not significantly positively associated with the engagement in walking for transport after adjustment for covariates.

### Commercial destinations

The positive associations between the availability of commercial destinations within walking distance and walking for transport are in line with the results of a recent systematic review and meta-analysis [14] which provided strong evidence on a positive relationship between the proximity to shops/commercial destinations and older adults’ total walking for transport.

In addition, former research showed that shops were the most frequently visited destinations of older adults [16] and also the most common destination older adults walk to [41]. Providing shops within walking distance could therefore encourage older adults to regularly walk to these destinations and thus integrate physical activity in daily routines.

The stronger associations observed for the availability of small stores and bakery in comparison to other commercial destinations investigated in this study suggest that walking for transport may be especially convenient for small errands where people do not have to carry heavy loads.

Concerning gender differences, a Japanese study found that the access to shops was significantly positively associated with walking for transport among women but not among men [19]. Contrary to these findings, this study showed that the availability of each of the investigated commercial destinations, except for the drugstore, was significantly positively associated with walking for transport in men but only small stores were significantly positively associated with walking for transport in women. According to these results, this study suggests that the availability of commercial destinations within walking distance may be especially important for walking for transport among older men living in smaller communities with less than 100,000 inhabitants. But the results have to be interpreted with caution since most of the point estimates of men are included in the respective confidence intervals of women.

Future studies should particularly focus on the further exploration of potential gender differences with respect to these associations.

### Service providers

This study found that the proximity to a post office, bank/credit union, salon/barber shop or laundry/cleaner’s was associated with higher odds of engagement in walking for transport in older adults. These findings are in agreement with the results of former studies which showed that a larger distance to the nearest bank [42] and the nearest post office [17] was negatively associated with walking for transport. A study from Nathan et al. [37] did not find an association between the proximity to financial services (i.e., bank, post office) and the engagement in some walking but showed a positive association between the proximity to general services (i.e., hairdresser or pharmacy) and the engagement in some walking. However, all these studies were conducted in urban areas outside of Europe and used methodologies that are different to this study, which limits the comparability.

It was somewhat surprising that the availability of a laundry/cleaner’s within a 20-min walk from home was significantly positively associated with walking for transport in older adults. Since the laundry/cleaner’s was one of the least commonly available destinations in the study region and people in this region usually wash their textiles at home, this finding should be regarded with caution.

### Eating places

The results of this study support previous findings from Hong Kong and the USA [15, 43] indicating a positive association of the availability of a restaurant with neighborhood-walking for transport and active transport among older adults. Furthermore, the present study adds some evidence on a positive relationship between the proximity to cafés and the engagement in walking for transport. Overall, this study suggests that the availability of eating places like cafés and restaurants may be also important for older adults’ walking for transport in smaller communities with less than 100,000 inhabitants. Securing the availability of these destinations may be also an opportunity to support older adults’ social participation which is another important factor for healthy aging [44].

### Health services

Former studies investigating the relationship between the availability of health services and the engagement in walking yielded mixed results. For example, a study from Hong Kong [15] showed that the diversity of health services was significantly positively associated with neighborhood walking for transport but not with overall walking for transport, and the prevalence of health services was not associated with any of these walking for transport outcomes. A study from Canada [42] examined the association between the distance to the nearest pharmacy and the nearest health facility and did not find any significant association with walking for transport. Nathan et al. [37] found a positive association between the availability of general services, including the availability of a pharmacy, and the engagement in some walking, but did not find a significant association between the availability of medical services (i.e., doctor, medical center) and some walking. A recent meta-analysis by Cerin et al. [14] confirmed the finding of a nil association.

Despite these findings, the results of this study showed that the availability of a physician and a pharmacy within walking distance from home were both significantly positively associated with the engagement in walking for transport. Furthermore, the strong associations found for the availability of a pharmacy suggests that the pharmacy may be one of the most important destinations for older adults’ walking for transport in small and medium-sized towns and rural communities. Planning age-friendly and activity-friendly environments, it is important to be aware that the need to take medicines on a regular basis as well as the proportion using prescribed medicines increases with age [45] so that the availability of a pharmacy as a daily need may be especially relevant to older adults.

### Recreational destinations

A number of studies have shown a positive association between the proximity to recreational facilities and walking for transport among older adults [20, 24–26] but little is known about the relationship of specific types of recreational destinations (e.g., recreation center, library, gym/fitness facility, park, cemetery) and the engagement in walking for transport. The findings of this study showed that the availability of each of the investigated recreational destinations within a 20-min walk from home was significantly positively associated with walking for transport. With respect to a walking distance of 10 min, only the availability of a library was associated with higher odds of engagement in walking for transport.

The observed positive associations of the proximity to a library and a park with the engagement in walking for transport are in accordance with previous findings from Procter-Gray et al. [17] but differ from the results obtained by Moniruzzaman et al. [42] who did not find an association between the distance to the nearest library and walking for transport.

Concerning the availability of a gym/fitness facility, King [18] investigated the association between the availability of exercise opportunities and tennis courts with walking for transport and did not find any associations. The results of this study, however, showed a positive relationship between the availability of a gym/fitness facility within a 20-min walk from home and the engagement in walking for transport. Additionally, these results suggest potential gender differences with respect to this association. In contrast to a Japanese study in which exercise facilities were significantly positively associated with walking for transport among women but not among men [19], this study showed that after adjustment for covariates the availability of a gym/fitness facility was significantly positively associated with walking for transport among men but not among women. However, these results have to be interpreted with caution since the point estimate of men is included in the confidence interval of women. Future studies are needed to further evaluate this association among men and women.

### Public transport

The bus stop was the most commonly available destination within walking distance in this study. However, it showed the weakest associations with walking for transport across all investigated destinations and these associations did not remain significant after adjustment for covariates. Even though these findings differ from former studies showing a significant positive association with walking for transport [20–23], the present results appear reasonable in the context of this specific study area. High car availability and low-frequency public transportation services in more rural areas make it unattractive to use public transport. Thus, it is not surprising that the availability of a bus stop was not significantly associated with walking for transport. Future studies should not only consider the availability of a transit stop but also the provision of public transport services.

### Strengths and limitations

This study is one of the first studies exploring the relationship between different types of destinations and walking for transport among older adults living in communities with less than 100,000 inhabitants. In this context, particular strengths are the wide variety of destinations assessed in this study and the focus on small and medium-sized towns and rural communities. Little is known about what is considered as a reachable walking distance for older adults living in these areas. It is therefore another strength that this study used two different distance categories (destinations within a 20-min and a 10-min walk from home) to define the availability of a destination and to investigate its association with walking for transport. It has been shown that even a low dose of moderate-to-vigorous physical activity of about 15 min per day (which could be reached, for example, by the engagement in walking for transport) was associated with health benefits like a reduced mortality in older adults [46]. Thus, it may be another strength that this study assessed the engagement in any walking for transport with a duration of at least 5 min vs. no walking for transport. A stronger association of walking for transport with the perceived availability of destinations than with objective measures of destination availability has been reported [14]. This finding supports the assumption that the perception of destinations may be closer related to the actual walking for transport than the objective presence of destinations. Considering this, it can be highlighted as a strength that this study used a perceived measure of destination availability.

However, this study is not without limitations. Firstly, the cross-sectional design of this study does not allow to draw conclusions on the causality of the observed associations. Secondly, this study only assessed the engagement in any walking for transport and did not differentiate between walking within and outside the neighborhood. Moreover, it did not assess the specific walking purposes of older adults’ walking for transport. Thus, it was not possible to derive any information on how much older adults actually walk within their neighborhood and to which specific destinations. Likewise, this study did not include any information on the geographical location of the destinations so that it was not possible to find out whether destinations are located closely to each other (e.g., in a shopping center) or not. Another limitation is that this study did not account for neighborhood self-selection. Lastly, it must be also considered that the high number of tests conducted within this study is related to a higher risk of spurious findings. However, the Bonferroni adjustment was applied to adjust for this kind of multiple testing.

Furthermore, it should be noted that the representativeness of the study population is somewhat limited. This study showed a smaller proportion of women, especially older women aged 80 years and over, in comparison with the general population of older adults residing in Lower Saxony [47] and also a smaller proportion of women living alone [48]. Additionally, a higher proportion of study participants self-rated their health as good or very good compared to the respective age groups of the German Ageing Survey [49]. However, the low risk-of-poverty rate [50] and the very low proportion of people born abroad [51] are comparable with those in the general population of Lower Saxony.

### Implications

This study underlines the importance of preserving local stores and services to foster older adults’ walking for transport in small and medium-sized towns and rural communities. It could be hypothesized that most frequently visited destinations may be especially important to integrate walking for transport in daily routines and thereby enhance the engagement in regular moderate physical activity in older adults. Although this study did not collect information on the frequency of visiting destinations, it provides some evidence that small stores, pharmacy, and bakery may be among the destinations most important for walking for transport in small and medium-sized towns and rural communities.

In addition, the findings suggest that in these smaller communities with less than 100,000 inhabitants it may be more important to have any destinations at all within walking distance than specific types of destinations. Consequently, providing nearly any type of destination within a 20-min walk from the older adults’ home may be beneficial for older adults’ walking for transport.

Urban and spatial planners together with economic actors such as the Chamber of Commerce and Industry should aim to support local businesses and thus make their communities more attractive for walking. This may be particularly challenging for smaller communities where many small, local stores are closing as they are unable to compete with larger stores in nearby cities or online businesses.

Nevertheless, municipalities could make an important contribution to change this situation and make local stores and services more attractive for both entrepreneurs and residents. Besides funding opportunities, municipalities could also raise more awareness among their residents to support local businesses.

Concerning the local planning of destinations, spatial scientists have already developed different concepts on how to secure the local supply and enhance the accessibility of destinations, either through centralization of destinations (e.g., revitalizing town centers) or decentralization. From a public health point of view and based on the findings of this study, it is highly recommended to strive for the availability of destinations within a 20-min walk from the older adults’ homes.

Furthermore, municipalities should also pay attention to the needs of older adults living far away from the municipality’s center and destinations. For this group of older adults, municipalities may consider establishing better public transport, e.g., improved bus services, which connect older adults to destinations and enable them to combine walking and public transport. Another option would be to promote ride sharing or similar informal transport services that are more spontaneous, cheaper, and probably faster than a bus service.

Concerning future research, further studies are needed which explicitly focus on the availability of destinations in small and medium-sized towns and rural communities and its potential influence on older adults’ walking for transport including frequency of visits. It is also important that more studies investigate these associations in specific subgroups of older adults to identify the special needs of vulnerable groups and better inform intervention strategies. Additionally, there is a need for qualitative studies to learn more about the underlying reasons of the associations between the availability of specific destinations and walking for transport in communities with less than 100,000 inhabitants and to better understand the walking behavior of older adults living in these areas.

## Conclusions

This study suggests that even in small and medium-sized towns and rural communities where people are used to make most of their journeys by car and drive until very old age, the availability of destinations within walking distance may encourage older adults to walk for transport.

The findings of this study underline the importance of securing local supply in small and medium-sized towns and rural communities from a public health perspective. Initiatives and policies should therefore aim to support local businesses and destinations to enable older adults to meet their daily needs independently and by foot. Providing destinations within walking distance of older adults’ homes should be put on top of the agenda to foster older adults’ walking for transport and thus enable active and healthy aging in place.

## Supplementary Information

**Additional file 1: Table S1.** Associations between the availability of destinations and walking for transport, stratified by walking aid use. **Table S2.** Associations between the availability of destinations and walking for transport, stratified by car availability.

## Data Availability

The datasets generated and analyzed during the current study are not publicly available due to data protection rules in Germany but are available for researchers who meet the criteria of the University of Bremen for access to confidential data from the corresponding author on reasonable request.
