# Supplementary material for: Destinations fostering older adults’ walking for transport: a cross-sectional study from Germany
Source: BMC Geriatr. 2022 Mar 17;22:219. doi: 10.1186/s12877-022-02896-w (PMC8928617; doi:10.1186/s12877-022-02896-w)
Supplement: Supplementary file 1 — Additional file 1: Table S1. Associations between the availability of destinations and walking for transport, stratified by walking aid use. Table S2. Associations between the availability of destinations and walking for transport, stratified by car availability. [file 12877_2022_2896_MOESM1_ESM.docx]

| **Table S1** Associations between the availability of destinations and walking for transport, stratified by walking aid use | | | | | | | | |
| --- | --- | --- | --- | --- | --- | --- | --- | --- |
|  | People using a walking aid (n=219)^a^ | | | | People not using a walking aid (n=1527)^a^ | | | |
|  | Crude OR | 95% CI^b^ | Adj. OR^c^ | 95% CI^b^ | Crude OR | 95% CI^b^ | Adj. OR^c^ | 95% CI^b^ |
| Commercial destinations |  |  |  |  |  |  |  |  |
| Bakery | 1.39 | 0.61-3.18 | 1.01 | 0.37-2.75 | 2.78 | 1.90-4.08 | 2.27 | 1.45-3.56 |
| Small grocery store | 1.72 | 0.76-3.92 | 1.36 | 0.52-3.52 | 1.92 | 1.34-2.76 | 1.58 | 1.08-2.32 |
| Supermarket | 1.50 | 0.66-3.42 | 1.07 | 0.40-2.86 | 1.99 | 1.39-2.86 | 1.52 | 1.01-2.31 |
| Drugstore | 1.73 | 0.63-4.74 | 1.41 | 0.45-4.37 | 2.14 | 1.41-3.24 | 1.71 | 1.10-2.66 |
| Small stores | 1.98 | 0.83-4.72 | 1.86 | 0.67-5.19 | 2.73 | 1.86-4.03 | 2.32 | 1.54-3.52 |
| Service providers |  |  |  |  |  |  |  |  |
| Post office | 2.32 | 1.00-5.40* | 2.13 | 0.81-5.60 | 2.17 | 1.51-3.12 | 1.76 | 1.19-2.61 |
| Bank/credit union | 2.15 | 0.93-4.98 | 1.94 | 0.71-5.32 | 2.10 | 1.46-3.01 | 1.69 | 1.14-2.50 |
| Salon/barber shop | 2.13 | 0.93-4.88 | 1.96 | 0.73-5.26 | 2.27 | 1.58-3.27 | 1.87 | 1.26-2.79 |
| Laundry/cleaner's | 2.88 | 0.97-8.56 | 2.83 | 0.83-9.69 | 2.03 | 1.36-3.04 | 1.62 | 1.06-2.49 |
| Eating Places |  |  |  |  |  |  |  |  |
| Café | 1.80 | 0.79-4.11 | 1.37 | 0.50-3.73 | 2.17 | 1.51-3.12 | 1.73 | 1.15-2.60 |
| Restaurant | 1.39 | 0.61-3.17 | 0.94 | 0.34-2.62 | 2.04 | 1.42-2.93 | 1.56 | 1.05-2.34 |
| Health services |  |  |  |  |  |  |  |  |
| Physician | 1.57 | 0.69-3.59 | 1.38 | 0.50-3.80 | 2.21 | 1.54-3.19 | 1.78 | 1.19-2.66 |
| Pharmacy | 2.23 | 0.97-5.11 | 1.76 | 0.65-4.77 | 2.46 | 1.70-3.54 | 2.00 | 1.34-2.99 |
| Recreational destinations |  |  |  |  |  |  |  |  |
| Recreation center | 2.07 | 0.88-4.89 | 2.12 | 0.76-5.93 | 1.81 | 1.26-2.60 | 1.50 | 1.02-2.22 |
| Library | 1.80 | 0.73-4.45 | 1.66 | 0.55-5.02 | 2.29 | 1.54-3.41 | 1.96 | 1.28-3.00 |
| Gym/fitness facility | 1.74 | 0.73-4.11 | 1.77 | 0.66-4.78 | 1.78 | 1.24-2.56 | 1.45 | 0.98-2.14 |
| Park | 1.37 | 0.61-3.11 | 1.42 | 0.53-3.79 | 2.22 | 1.54-3.20 | 1.93 | 1.31-2.84 |
| Cemetery | 1.72 | 0.73-4.05 | 1.94 | 0.71-5.34 | 1.58 | 1.10-2.26 | 1.45 | 0.99-2.12 |
| Public transport |  |  |  |  |  |  |  |  |
| Bus stop | 1.82 | 0.74-4.44 | 1.68 | 0.61-4.68 | 1.60 | 1.04-2.46 | 1.29 | 0.81-2.05 |
| OR=Odds Ratio; CI=Confidence Interval; Adj.=Adjusted; ^a^ Separate models were computed for all listed destinations. ^b^ Confidence intervals were adjusted using the Bonferroni method. ^c^ OR were adjusted for gender, age, education, income, living situation, area of residence, bicycle availability, walking infrastructure and connectivity. * After the Bonferroni adjustment the p-value was ≥ 0.0026 indicating a non-significant result. | | | | | | | | |

| **Table S2** Associations between the availability of destinations and walking for transport, stratified by car availability | | | | | | | | |
| --- | --- | --- | --- | --- | --- | --- | --- | --- |
|  | People having always access to a car (n=1557)^a^ | | | | People having limited or no access to a car (n=167)^a^ | | | |
|  | Crude OR | 95% CI^b^ | Adj. OR^c^ | 95% CI^b^ | Crude OR | 95% CI^b^ | Adj. OR^c^ | 95% CI^b^ |
| Commercial destinations |  |  |  |  |  |  |  |  |
| Bakery | 2.56 | 1.78-3.68 | 1.90 | 1.23-2.92 | 3.22 | 1.04-9.94 | 2.72 | 0.65-11.39 |
| Small grocery store | 1.98 | 1.40-2.81 | 1.58 | 1.09-2.29 | 1.88 | 0.68-5.20 | 1.64 | 0.48-5.54 |
| Supermarket | 1.93 | 1.37-2.74 | 1.37 | 0.91-2.05 | 2.51 | 0.89-7.10 | 2.25 | 0.65-7.83 |
| Drugstore | 2.05 | 1.38-3.06 | 1.54 | 1.00-2.37* | 3.94 | 1.09-14.31 | 3.49 | 0.80-15.26 |
| Small stores | 2.67 | 1.84-3.88 | 2.17 | 1.45-3.25 | 3.09 | 1.07-8.90 | 3.39 | 0.95-12.05 |
| Service providers |  |  |  |  |  |  |  |  |
| Post office | 2.14 | 1.51-3.03 | 1.69 | 1.15-2.48 | 4.13 | 1.39-12.28 | 4.97 | 1.29-19.12 |
| Bank/credit union | 2.13 | 1.50-3.02 | 1.66 | 1.13-2.44 | 2.68 | 0.95-7.55 | 2.93 | 0.71-12.04 |
| Salon/barber shop | 2.25 | 1.59-3.19 | 1.81 | 1.22-2.67 | 3.00 | 1.06-8.52 | 3.25 | 0.91-11.55 |
| Laundry/cleaner's | 2.17 | 1.46-3.23 | 1.65 | 1.08-2.53 | 3.19 | 0.92-11.05 | 3.05 | 0.72-12.92 |
| Eating Places |  |  |  |  |  |  |  |  |
| Café | 2.20 | 1.55-3.12 | 1.64 | 1.10-2.44 | 2.40 | 0.85-6.83 | 2.15 | 0.57-8.07 |
| Restaurant | 2.06 | 1.45-2.91 | 1.50 | 1.01-2.23 | 1.75 | 0.63-4.83 | 1.53 | 0.43-5.46 |
| Health services |  |  |  |  |  |  |  |  |
| Physician | 2.12 | 1.50-3.01 | 1.62 | 1.09-2.40 | 2.94 | 1.03-8.39 | 3.24 | 0.89-11.79 |
| Pharmacy | 2.35 | 1.66-3.33 | 1.83 | 1.24-2.72 | 3.67 | 1.28-10.52 | 4.86 | 1.23-19.19 |
| Recreational destinations |  |  |  |  |  |  |  |  |
| Recreation center | 1.92 | 1.36-2.73 | 1.57 | 1.07-2.30 | 1.99 | 0.69-5.78 | 1.74 | 0.48-6.28 |
| Library | 2.21 | 1.51-3.23 | 1.85 | 1.22-2.79 | 2.67 | 0.83-8.61 | 2.88 | 0.71-11.67 |
| Gym/fitness facility | 1.79 | 1.27-2.54 | 1.38 | 0.94-2.01 | 3.39 | 1.11-10.35 | 3.31 | 0.91-12.03 |
| Park | 2.17 | 1.53-3.07 | 1.80 | 1.23-2.62 | 2.11 | 0.76-5.86 | 2.68 | 0.78-9.19 |
| Cemetery | 1.74 | 1.23-2.46 | 1.53 | 1.06-2.23 | 1.42 | 0.49-4.10 | 1.51 | 0.43-5.27 |
| Public transport |  |  |  |  |  |  |  |  |
| Bus stop | 1.72 | 1.15-2.57 | 1.29 | 0.83-2.00 | 1.56 | 0.48-5.08 | 1.29 | 0.31-5.32 |
| OR=Odds Ratio; CI=Confidence Interval; Adj.=Adjusted; ^a^ Separate models were computed for all listed destinations. ^b^ Confidence intervals were adjusted using the Bonferroni method. ^c^ OR were adjusted for gender, age, education, income, living situation, area of residence, use of a walking aid, bicycle availability, walking infrastructure and connectivity. * After the Bonferroni adjustment the p-value was ≥ 0.0026 indicating a non-significant result. | | | | | | | | |
